# Periodontal Bone-Ligament-Cementum Regeneration via Scaffolds and Stem Cells

**DOI:** 10.3390/cells8060537

**Published:** 2019-06-04

**Authors:** Jin Liu, Jianping Ruan, Michael D. Weir, Ke Ren, Abraham Schneider, Ping Wang, Thomas W. Oates, Xiaofeng Chang, Hockin H. K. Xu

**Affiliations:** 1 Key Laboratory of Shannxi Province for Craniofacial Precision Medicine Research, College of Stomatology, Xi’an Jiaotong University, 98 XiWu Road, Xi’an 710004, China; jliu2@umaryland.edu (J.L.); ruanjp@xjtu.edu.cn (J.R.); 2 Clinical Research Center of Shannxi Province for Dental and Maxillofacial Diseases, College of Stomatology, Xi’an Jiaotong University, 98 XiWu Road, Xi’an 710004, China; 3 Department of Advanced Oral Sciences and Therapeutics, University of Maryland Dental School, Baltimore, MD 21201, USA; michael.weir@umaryland.edu (M.D.W.); dentistping@gmail.com (P.W.); toates@umaryland.edu (T.W.O.); 4 Department of Neural and Pain Sciences, School of Dentistry, & Program in Neuroscience, University of Maryland, Baltimore, MD 21201, USA; kren@umaryland.edu; 5 Department of Oncology and Diagnostic Sciences, University of Maryland School of Dentistry, Baltimore, MD 21201, USA; schneider66@umaryland.edu; 6 Member, Marlene and Stewart Greenebaum Cancer Center, University of Maryland School of Medicine, Baltimore, MD 21201, USA; 7 Center for Stem Cell Biology & Regenerative Medicine, University of Maryland School of Medicine, Baltimore, MD 21201, USA

**Keywords:** periodontal regeneration, bone-PDL-cementum, scaffolds, stem cells, growth factors, tissue engineering

## Abstract

Periodontitis is a prevalent infectious disease worldwide, causing the damage of periodontal support tissues, which can eventually lead to tooth loss. The goal of periodontal treatment is to control the infections and reconstruct the structure and function of periodontal tissues including cementum, periodontal ligament (PDL) fibers, and bone. The regeneration of these three types of tissues, including the re-formation of the oriented PDL fibers to be attached firmly to the new cementum and alveolar bone, remains a major challenge. This article represents the first systematic review on the cutting-edge researches on the regeneration of all three types of periodontal tissues and the simultaneous regeneration of the entire bone-PDL-cementum complex, via stem cells, bio-printing, gene therapy, and layered bio-mimetic technologies. This article primarily includes bone regeneration; PDL regeneration; cementum regeneration; endogenous cell-homing and host-mobilized stem cells; 3D bio-printing and generation of the oriented PDL fibers; gene therapy-based approaches for periodontal regeneration; regenerating the bone-PDL-cementum complex via layered materials and cells. These novel developments in stem cell technology and bioactive and bio-mimetic scaffolds are highly promising to substantially enhance the periodontal regeneration including both hard and soft tissues, with applicability to other therapies in the oral and maxillofacial region.

## 1. Introduction

Periodontitis is a widespread infectious oral disease, characterized by irreversible damage in the tooth-supporting tissues, which include the alveolar bone, periodontal ligament (PDL), and cementum. This eventually leads to tooth loss with serious functional and aesthetic problems for the patients [1]. An epidemiological survey has suggested that > 50% of all adults in the world are affected by periodontal diseases. Furthermore, periodontal disease occurrence is increasing with time; in the 10-year period from 2005 to 2015, the prevalence rates have risen rapidly compared to earlier periods [2]. Periodontal disease pathogenesis involves complicated interactions between the host’s immune response with the microbial colony in the periodontal pocket, as well as other factors including smoking and genetics. The sub-gingival biofilms on the teeth, which are extracted from patients with periodontitis, consist of an aggregation of bacterium that is attached and embedded into a matrix on the tooth surface. The inflammatory reaction of the periodontal tissues has been verified by the large numbers of leukocytes which are mobilized in the vicinity of inflammation [3]. Furthermore, periodontitis is related to the occurrence and development of a number of other systemic diseases, such as cardiovascular diseases, cancer, rheumatoid arthritis, obesity and diabetes [4,5,6,7]. The diagnosis for the damage of the periodontal tissue includes clinical symptoms (gingival bleeding, bone resorption, periodontal pocket formation, attachment loss), X-ray and cone-beam computed tomography (CBCT) [8], and genetic polymorphisms [9].

The aim of periodontal treatment is to control the infection and reconstruct the structures and functions of the periodontal tissues [10]. Challenges remain in regenerating the periodontal apparatus with the formation of the bone-PDL-cementum complex simultaneously [11]. The osteogenic process might slightly precede the differentiation of cementum and fibers. Then the oriented PDL would need to be attached firmly to the newly-formed cementum and the alveolar bone, which is especially challenging to achieve in the laboratory [12].

Several meritorious articles and reviews were published on the regeneration of periodontal tissues. For instance, one review described the application of stem cells in periodontal tissue regeneration [13]. Another review discussed the guided tissue regeneration (GTR) and various GTR membranes [14]. Another review summarized natural grafts and synthetic biomaterial scaffolds for periodontal and bone repairs [15]. However, a systematic review is still needed on the new developments in regenerating all three types of periodontal tissues and the simultaneous regeneration of the entire bone-PDL-cementum complex. The present review focuses on the cutting-edge research on periodontal regeneration. It includes the mechanisms of regeneration for bone, PDL and cementum; the use of transplanting exogenous stem cells and mobilization of endogenous stem cells from their niches; the development of absorbable, injectable and bio-printed materials; gene therapy and layered biomimetic technologies for bone-PDL-cementum regeneration. It is highly important to translate and apply new research and technologies to clinical treatments. The novel developments via stem cells and bio-mimetic scaffolds are promising to substantially enhance the periodontal bone-ligament-cementum regeneration including both hard and soft tissues, with applicability to other therapies in the oral and maxillofacial region.

## 2. Bone Regeneration

Bone loss is a major hallmark of periodontitis. Pathogenic microorganisms in the biofilm, genetic factors, and environmental issues such as tobacco use, can all contribute to periodontitis and bone loss. Losing the supporting bone around a tooth results in tooth movement and dislocation, eventually leading to tooth loss [16]. Various techniques have been developed to enhance the osteogenesis process, including bone grafts, [17], scaffolds [18], stem cells [19] and growth factors. Bone grafts can be conveniently divided into four groups [20,21]. First, autogenous bone grafts are generally viewed as the “gold standard” for bone replacement [22]. Clinical applications showed that new bone and new periodontal connective tissue attachment were obtained [23]. Second, tissue banks provide different types of allogeneic bone grafts [24], including freeze-dried bone allografts (FDBAs) and demineralized freeze-dried bone allografts (DFDBAs). Clinical trials showed that periodontal bone fillings of 1.3 to 2.6 mm were obtained. DFDBAs generated significantly more vital bone at 38.4%, compared to that via FDBAs at 24.6% [25]. Third, xenografts have been used, for example, Bio-Oss [26]. One study investigated the effects of employing titanium mesh in conjunction with Bio-Oss for localized alveolar ridge augmentation. Radiographic analyses showed that a 2.86 mm vertical and 3.71 mm buccolabial ridge augmentation was obtained, while histomorphometry analysis showed that 36.4% of the grafted region had new bone [15]. Forth, synthetic alloplastic materials have been developed, for example, hydroxyapatite (HA) [27,28,29], tricalcium phosphates (TCP) [30], a calcium-layered-polymer of polymethyl methacrylate and hydroxyethyl methacrylate (PMMA and HEMA polymer [31] and bioactive glass [32].

**Figure 1:**
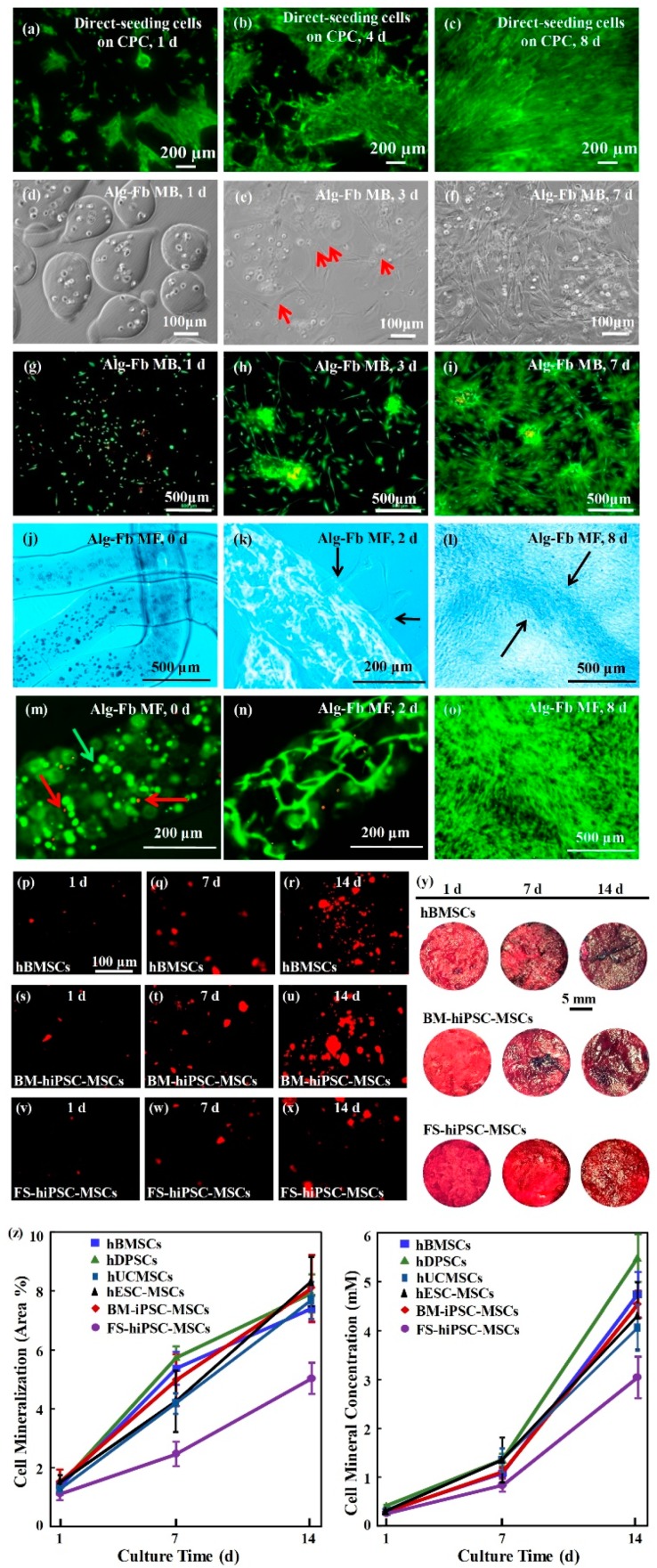
Methods of cell delivery via calcium phosphate cements (CPC). Live-dead staining of cell seeding on CPC (**a**–**c**); cell encapsulation in alginate-fibrinogen microbeads (Alg-Fb-MB) (**d**–**i**); cell encapsulation in alginate–fibrinogen microfibers (Alg-Fb-MaF) (**j**–**o**); synthesis of bone minerals by the encapsulated stem cells. Images of (**p**–**r**) hBMSCs, (**s**–**u**) BM-hiPSC-MSCs, and (**v**–**x**) FS-hiPSC-MSCs stained with Xylenol orange (images of hESC-MSCs, hUCMSCs, and hDPSCs were similar to those of hBMSCs). (**y**) Alizarin red S (ARS) staining of hBMSCs, BM-hiPSC-MSCs and FS-hiPSC-MSCs in calcium phosphate cements-cell encapsulating alginate-fibrin fibers (CPC-CAF) (images of hESC-MSCs, hUCMSCs, hDPSCs were similar to those of hBMSCs). (**z**) Xylenol orange mineral staining area and ARS mineral concentration produced by cells in CPC-CAF (mean ± s.d.; n = 6). (Adapted from References [39,41,45], with permission.).

**Figure 2:**
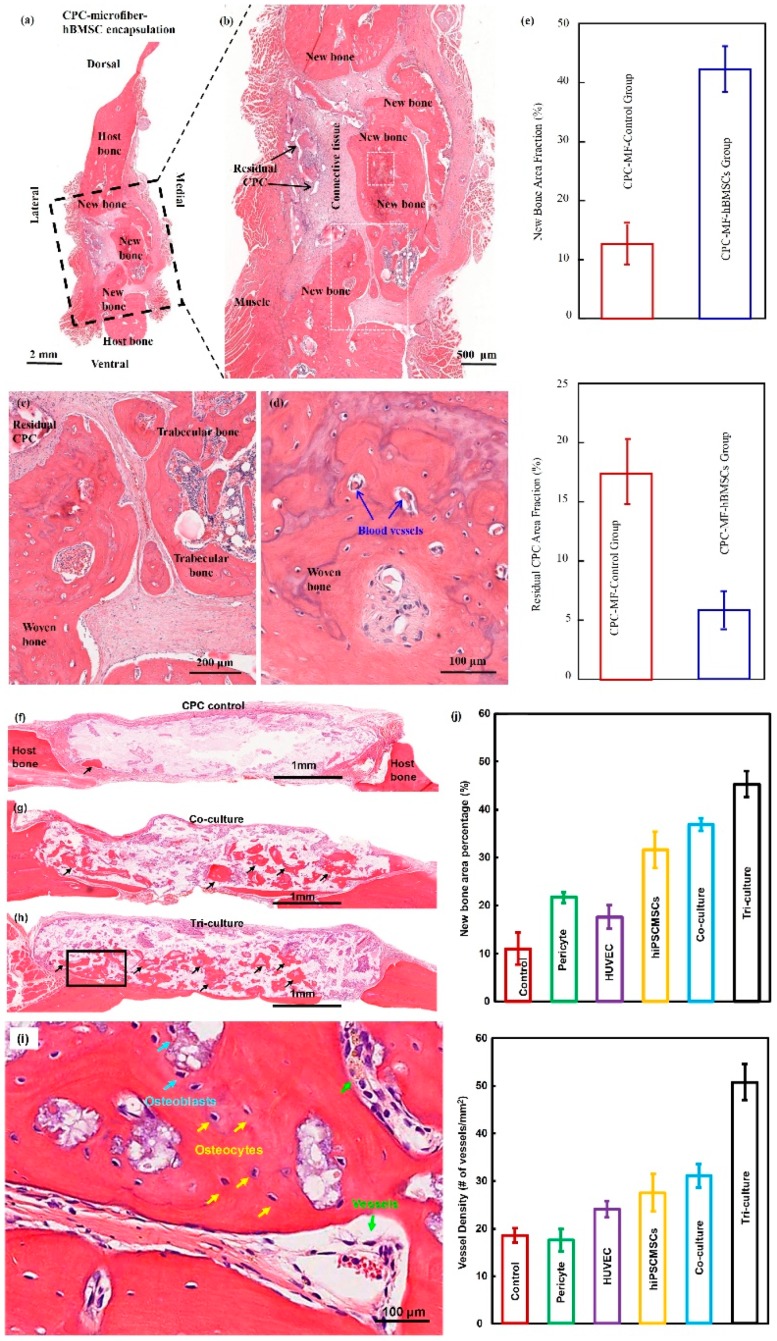
CPC-stem cells construct for bone regeneration. (**a**) Representative hematoxylin-eosin (HE) images of the CPC-MF-hBMSC group at 12 weeks post-surgery. Bone bridging was achieved in the critical-sized defects. The defect was closed with newly woven bone and trabecular bone. (**c**) and (**d**) were high magnification images of the dotted-line rectangle in (**b**); (**e**) quantification of new bone and residual CPC area fraction. (**f**–**h**) Representative HE images at 12 weeks. The cell-seeded groups showed more new bone than CPC control. The greatest amount of new bone was observed in the tri-culture group; (**i**) high magnification images of new bone from dotted rectangles in the tri-culture group (**h**). New blood vessels in the macropores of CPC scaffolds; (**j**) quantification of the new bone area and vessel density (adapted from References [37,41] with permission).

Injectable and absorbable scaffolds were developed for bone regeneration applications [33,34]. Among them, calcium phosphate cements (CPCs) consisted of calcium phosphate powders which were mixed with a liquid to form a paste [35,36]. The paste could be injected into the bone defect site to harden in situ to form a scaffold, through a dissolution-precipitation reaction at 37 °C [37]. Cell seeding onto the porous CPC scaffold yielded a relatively poor seeding efficacy and mediocre cell penetration into the scaffold (Figure 1a–c) [38]. It was not feasible to directly mix the cells with the paste due to the mixing stresses, ionic exchanges, and pH variations during the CPC paste setting were harmful to the cells. Therefore, a resorbable and injectable alginate-microfibers/microbeads (Alg-MB/MF) delivery system for stem cells was developed, which protected the encapsulated stem cells during the CPC paste blending and injection [39]. It supported cell health, proliferation and differentiation, with microbeads degrading at 3–4 days and releasing the encapsulated cells (Figure 1d–o) [40,41]. In a recent study [42], six types of stem cells: human bone mesenchymal stem cells (hBMSCs), human dental pulp stem cells (hDPSCs) [43,44], human umbilical cord MSCs (hUCMSCs), MSCs derived from embryonic stem cells (hESC-MSCs), human induced pluripotent stem cell-MSCs derived from bone marrow (BM-hiPSC-MSCs) and from foreskin (FS-hiPSC-MSCs), were encapsulated in hydrogel microfibers and microbeads inside an injectable CPC. All the above cells proliferated and osteodifferentiated well, exhibiting high expressions of osteogenic genes at 7 days. Cell-synthesized bone matrix minerals were enhanced with increasing culture time, indicating excellent bone regeneration capability similar to the gold-standard hBMSCs (Figure 1p–z) [45]. Next, by implanting the hBMSC-encapsulated Alg-MB-CPC paste into a bone defect for bone regeneration in rats, the construct showed a potent capability for new bone formation. At 12 weeks, an osseous bridge was formed in the bone defect, having an area fraction for the new bone of 42.1% ± 7.8%, which was three-fold greater than that of the control group (Figure 2a–e) [41]. Therefore, the absorbable, injectable, load-bearing, stem cell-MB/MF-CPC construct was promising for cell delivery to greatly enhance bone regeneration in periodontal repairs [37]. Other hybrid poly (ethylene glycol)-co-peptide hydrogels could be tailored to the requirements of in situ gelation, which provided an alternative for injectable applications. This novel hydrogel has promising applications in endogenous regeneration, representing a more updated therapeutic strategy [46].

Furthermore, it was important to establish vascularization in periodontal regeneration [47,48]. Recently, a tri-culture system was formulated that included hiPSC-MSCs, human umbilical vein endothelial cells (HUVECs) and pericytes to provide pre-vascularization to CPC scaffold [49]. Vessel-like structures were successfully formed in both the co-cultured and tri-cultured groups in vitro. In addition, much higher angiogenic and osteogenic marker expressions, as well as bone matrix mineralization, were obtained. A cranial bone defect model in rats was used and after 12 weeks, the tri-culture group generated much greater new bone amount (45%, 4.5 folds) as well as new blood vessel density (50%, 2.5 folds), when compared with CPC control. The area fraction of the newly-formed bone and the blood vessel density in the tri-culture constructs were approximately 1.2-fold and 1.7-fold those of the co-culture group, respectively (Figure 2f–j) [41].

In addition, novel nanomaterials were developed in conjunction with other scaffold materials and biologics to enhance periodontal tissue regeneration [50,51,52,53,54]. BMSCs were transfected with BMP-7, seeded on nHA/PA porous scaffolds, and then placed in vivo using a rabbit mandibular defect model [55,56]. The scaffolds having BMP-7-transfected MSCs demonstrated a faster response than MSCs/scaffolds and pure nHA/PA scaffolds. Therefore, this study showed the importance of the factors and cells in facilitating bone regeneration. In a separate study, gold nanoparticles (GNPs) were incorporated into CPC [57]. This improved cell adhesion, proliferation and osteogenic induction on CPC. In addition, the released GNPs were internalized by hDPSCs and thus enhanced the expression of alkaline phosphatase (ALP) at 7 days (about 3 folds), osteogenic gene at 14 days (about 2-3 folds) and cell mineral at 14 days (about 5 folds). Therefore, nanoparticles were promising to modify the scaffolds and work together with other bioactive additives to enhance bone regeneration in periodontal repairs. 

## 3. PDL Regeneration

The PDL fibers connect the cementum on the tooth root surface to the alveolar bone and fix the tooth in the alveolar socket to attenuate the occlusal stresses. The regeneration of PDL is an important requirement for periodontal regeneration. The ideal outcome would be that the regenerated highly-organized collagen fibers could re-insert perpendicularly and firmly attached to the regenerated cementum and new bone [58].

Inflammation in the periodontal pocket can change the cell biology in the pathological periodontium. Once it is damaged, the periodontium has an only limited capacity for regeneration, which relies on the availability of MSCs. Several types of MSCs remain and are responsible for tissue homeostasis, serving as a source of renewable progenitor cells to generate other required cells throughout adult life. In addition, studies to date have shown that periodontal stem cells can be transplanted into periodontal defects with no adverse immunologic or inflammatory consequences. Therefore, periodontal regeneration relies on the successful recruitment of locally-derived renewable progenitor cells to the lesion site for tissue homeostasis and subsequent differentiation into PDL, cementum and bone-forming cells [59]. New PDL-like tissues were successfully formed via the delivery of stem cells to the defect sites [52,60], including the delivery of periodontal ligament stem cells (PDLSCs) [13,61] BMMSCs [62], adipose-derived stem cells (ADSCs) [63], and induced pluripotent stem cells (iPSCs) [64]. PDLSCs were cultured and osteogenically induced and then seeded on a biphasic calcium phosphate scaffold (BCP) [61]. Then the PDLSC-seeded scaffolds were transplanted into six dogs. The results showed that the transplantation of PDLSC-seeded BCP promoted effective periodontal regeneration, including new bone formation and PDL with reorganized and reborn collage fibers inserting into adjacent cementum and bone at the right angle, along with abundant blood vessels at 12 weeks. Therefore, PDLSC-seeded scaffolds were a promising method for periodontal regeneration.

**Figure 3:**
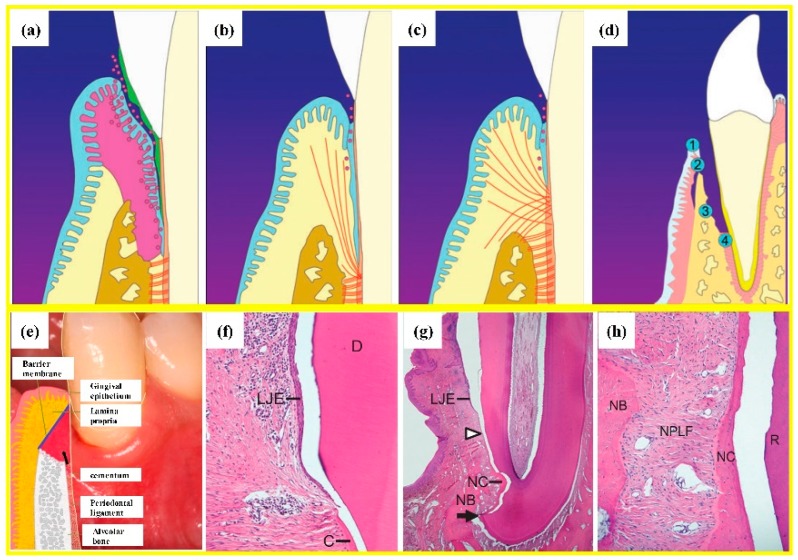
Periodontal regeneration. (**a**) inflamed soft tissue and bone resorption in periodontitis; (**b**) periodontal long junctional epithelium (LJE) repair; (**c**) ideal periodontal regeneration; (**d**) schematic of the four compartments from which cells could grow into periodontal wound and repopulate the root surface after periodontal treatment: ➀ oral gingival epithelium; ➁ gingival connective tissue; ➂ bone; ➃ PDL; (**e**) schematic of guided tissue regeneration (GTR). (**f**) optical micrograph shows LJE ending at the coronal-most end of the regenerated cementum (C) and dentin (D); (**g**) LJE and partial periodontal regeneration, indicated by the formation of new cementum (NC) and new bone (NB). The arrowhead indicates the apical end of the junctional epithelium, whereas the arrow shows the apical border of the defect. (**h**) optical micrograph showing periodontal regeneration, with the formation of new PDL fibers (NPLF) attaching to both NB and NC. R: root (adapted from References [65,66], with permission).

However, several implantations of bone substitute materials into the periodontal wounds produced a long junctional epithelium (LJE) but did not have the ability to regenerate a real periodontium [65]. As shown in Figure 3, the formation of an LJE only reduced the periodontal pocket depth but had no regeneration of PDL fibers (Figure 3b). In contrast, ideal periodontal tissue regeneration, needed well-organized fibers attaching to the adjacent new cementum and bone (Figure 3c) [66,67]. A barrier membrane was used to maintain the space between the defect and the root surface to enhance the proliferation of PDLSC and the synthesis of both PDL and bone (Figure 3e) [66]. Animal studies showed periodontal regeneration histologically (Figure 3f–h) [65]. Therefore, GTR could guide the soft tissue regeneration without down-growth into the bone defects, thereby promoting the regeneration of the periodontium [68].

Non-resorbable materials were prone to be exposed to the oral environment to increase the risk of post-operative infection [69]. Several subjects were available for the examination of vertical clinical attachment level (CAL-V) gain at 12 and 120 months after GTR therapy. 3.4 ± 1.0 mm and 1.5 ± 1.2 mm CAL-V were gained for the non-resorbable barrier group at 12 and 120 months, respectively. 3.3 ± 1.6 mm and 3.5 ± 2.5 mm were gained for the bioabsorbable barrier group at 12 and 120 months, respectively [70]. Another study showed that the mean probing depths (PD) reduction at 9 months was 5.2 ± 3.9 mm for bioabsorbable sites, and 5.5 ± 3.0 mm for non-resorbable sites. The CAL gain at 9 months was 5.9 ± 3.3 mm and 5.5 ± 3.4 mm, for resorbable and non-resorbable groups, respectively [71]. Therefore, there was no significant difference in the short term (9 or 12 months), but long-term (120 months) observation showed that the absorbable group could obtain better CAL-V than the non-resorbable group (2.34 fold). Therefore, absorbable materials were worth to be investigated. First, PDLSCs and gingival mesenchymal stem cells (GMSCs) were encapsulated in a novel arginine-glycine-aspartic acid tripeptide (RGD)-coupled alginate microencapsulation system to test the bone regeneration capacity (BRC) [72]. The microencapsulation scaffolds enhanced the osteogenic differentiation in vitro by expression of osteogenic markers runt-related transcription factor 2 (Runx2), ALP, and osteocalcin (OCN). Critical-sized calvarial defects of 5 mm were created in immunocompromised mice, and the cell-RGD-alginate construct was transplanted. New PDL fibers and bone were successfully formed at 8 weeks [72,73]. Second, the mixture of chitosan (CS), poly (lactic-co-glycolic acid) (PLGA), and silver nanoparticles was investigated for periodontal tissue engineering applications [74,75]. This method yielded cell mineralization and the expression of osteogenic genes in vitro. Meanwhile, the bone density of the experimental group was higher than that in the control group after implanting the materials into the mandible in vivo [76]. Third, a sequential collagen self-assembly method was combined with diffusion gradients in mineral formation to yield multiphasic collagen scaffolds that had interconnectivity and macroporosity between the layers [77]. The scaffolds had mineralization in the layers, wherein the mineralized collagen fibrils had intrafibrillar and oriented minerals that resembled bone. In addition, the non-mineralized fibrils were inserted into the mineralized layer to create the mechanical interlock and cohesion. Hence, these absorbable materials could avoid the need for a second surgery and the delayed wound healing process.

Another study investigated the effect of biomimetic electrospun fish collagen/bioactive glass/chitosan composite nanofiber membrane (Col/BG/CS) on periodontal regeneration [78]. This composite nano-membrane possessed a biomimetic microstructure with excellent hydrophilicity (the contact angle was 12.83 ± 3°) and a relatively high tensile strength (13.1 ± 0.43 MPa). Compared to the pure fish collagen membrane, the composite membrane exhibited an antibacterial function against *Streptococcus mutans*. The composite membrane not only promoted cell growth and osteogenic gene expression of hPDLCs but also elevated the expression of Runx2 and OPN proteins in vitro. Animal tests in dogs verified this nano-membrane’s ability to promote PDL and new bone formation in class II (Glickman’s) furcation defects. This novel membrane had a high level of macroporosity and a high surface area to promote cell–cell and cell–matrix interactions, with adequate tensile strength, antibacterial properties and osteogenic properties to enhance periodontal regeneration.

## 4. Cementum Regeneration

The cementum occurred as a thin acellular layer around the root neck, with thicker cellular cementum covering the lower part of the root up to the apex [79,80,81]. Hertwig’s epithelial root sheath (HERS) cells were suggested to secrete acellular cementum in the initial stage of cementogenesis. Later on, cellular and reparative cementum was produced by the dental follicle-derived cementoblast. Several diseases such as periodontitis usually affected the acellular cementum. However, the predictability and quality of cementum regeneration in an everyday clinical situation appeared to be low. Ideally, the regenerated cementum should closely resemble the acellular extrinsic fiber cementum (AEFC), because it contributed most to the attachment function. In most periodontal regeneration studies, the quality of the attachment function was questionable, because the newly-formed cementum was cellular intrinsic fiber cementum (CIFC), instead of the desired AEFC. The numerical density of the inserting fibers in CIFC was low, and the interfacial tissue bonding appeared to be weak [79,80,81].

Several cementum-specific proteins were shown to promote new cementum and bone formation for the damaged periodontal tissues [82]. These proteins included cementum-derived growth factor (CDGF), cementum attachment protein (CAP) and cementum protein-1 (CEMP1). They could induce several signaling pathways associated with mitogenesis, increase the concentration of cytosolic Ca^2+^, activate the protein kinase C cascade, and promote the migration and preferential adhesion of progenitor cells. These actions could result in the cementoblast and osteoblast differentiation and the production of a mineralized extracellular matrix resembling the cementum [82]. Indeed, the addition of CEMP1 to the 3D PDLCs cultures increased the ALP specific activity by 2-fold and induced the expression of cementogenic and osteogenic markers, forming new tissues that mimicked bone and cementum [83]. 

The stem cells in the PDL, gingiva, and alveolar bone served as sources for cementoblast progenitors [84], producing cementum-specific markers and cementum-like mineralized nodules in culture [85]. Indeed, PDLSCs, stem cells from the dental follicle (DFSCs), and ADSCs were all able to differentiate into cementoblasts and regenerate the periodontium to form cementum-like tissue, as well as PDL fibers and periodontal vessel regeneration in vivo [86,87]. In another study, DFCSs were combined with the treated dentin matrix (TDM) and implanted subcutaneously into the dorsum of mice. Histological examination revealed a whirlpool-like alignment of the DFCs in multiple layers that were positive for collagenase I (COLI), integrin β1, fibronectin and ALP, suggesting the formation of a rich extracellular matrix. TDM could induce and support DFCSs to develop new cementum-periodontal complexes and dentin-pulp like tissues, implying successful root regeneration [88]. Therefore, transplanting these types of stem cells into periodontal defects would be an effective technique for cementum regeneration [89].

**Figure 4:**
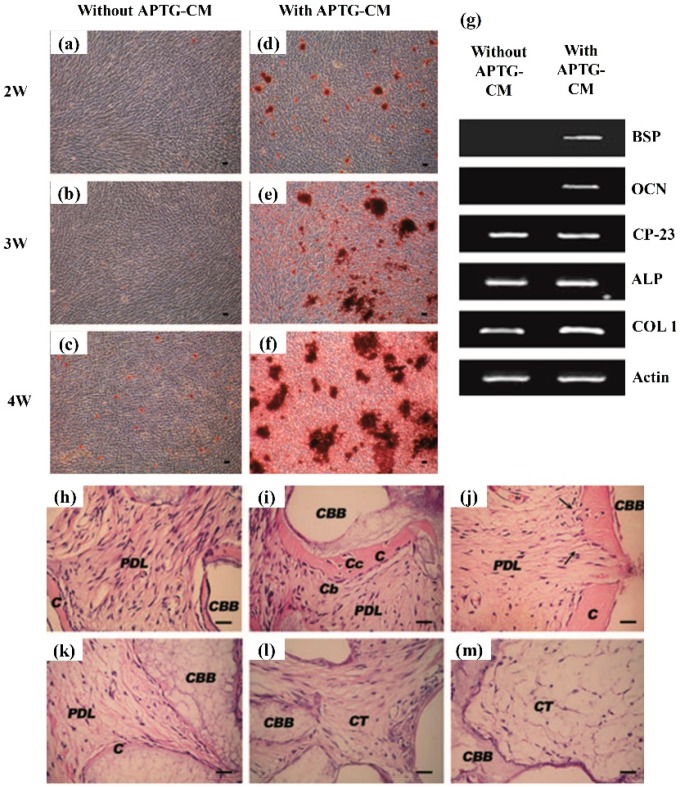
Apical tooth germ cell conditioned medium (APTG-CM) enhanced differentiation of periodontal ligament stem cells (PDLSCs) into cementum/periodontal ligament-like tissues. (**a**–**c**) PDLSCs in differentiation (a-modified eagle medium (a-MEM) with 10% fetal bovine serum (FBS), 100 μg/mL penicillin and 100 μg/mL streptomycin, 50 μg/mL of ascorbic acid and 2 mM sodium β-glycerophosphate) without APTG-CM had little mineral. (**d**–**f**) ultures with APTG-CM had substantial minerals. (**g**) Gene expression of PDLSCs co-cultured with APTG-CM. Osteocalcin (OCN), bone sialoprotein (BSP) and cementum-derived protein-23 (CP-23) expressions served as markers for cementoblast differentiation. At 21 days co-culture with APTG-CM, there were elevated expressions of OCN and BSP mRNA in the induced PDLSCs. Untreated PDLSCs lacked OCN and BSP expressions. (**h**) PDLSCs co-cultured with APTG-CM generated cementum-like minerals (C) on bovine bone (CBB) powders and PDL-like collagen fibers (PDL) connected with the new cementum. (**i**) There were cementoblast-like cells (Cb) at the cementum–PDL interface and cementocyte-like cells (Cc) in the mineral matrix. (**j**) Collagen bundles (arrows) were attached to cementum. (**k**, **l**) Untreated PDLSCs had little cementum or PDL-like structures. (**m**) No mineralization or PDL-like tissues were seen in CBB alone. Scale bars = 100 μm. APTG-CM: CT: connective tissue (adapted from Reference [90], with permission).

Furthermore, co-culturing several types of cells could also promote cementoblast differentiation. The biological effects of the conditioned medium from the developing apical tooth germ cells (APTG-CM) on the differentiation and cementogenesis of PDLSCs were investigated. The PDLSCs were cultured together with APTG-CM and demonstrated properties of the cementoblast lineages. This included morphological changes, greater proliferation, elevated ALP activity, and the expression of cementum-related genes and mineral nodules (Figure 4a–g). An immunocompromised mice model was tested, and after transplantation in vivo, the induced PDLSCs demonstrated tissue-regenerative ability and generated new cementum and periodontal ligament-like structures. This structure contained a layer of cementum-like mineralized tissues with periodontal ligament-like collagen fibers which were attached to the new cementum. In comparison, the untreated PDLSC transplant control generated only connective tissues (Figure 4h–m) [90]. Therefore, APTG-CM demonstrated the capability of providing a cementogenic microenvironment and promoting the differentiation of PDLSCs into the cementoblastic lineage, thereby enhancing periodontal tissue engineering.

The “cell sheet” technique was based on culturing the cells in hyper-confluency until they established extensive cell-cell interactions and produced their own extracellular matrix by forming a cell sheet [91]. The cells in the hDPSC cell sheets could survive for at least 96 h and retain their stemness and their osteogenic differentiation potential. Hence, cell sheets of hDPSCs could be employed as a natural three-dimensional structure for treating bone loss [92]. Similarly, canine PDL cells were seeded on temperature-responsive culture dishes in a complete medium supplement for 3 days and then in an osteo-inductive culture medium for an additional 2 days to reach over-confluency. The PGA sheets and the cell sheets were subsequently obtained by peeling them off from the dishes with forceps [93]. This method was repeated twice more, which finally generated the PDL cell sheets. The three-layered PDL cell sheets, supported by the PGA sheets, were placed on the exposed tooth root surfaces. Then the three-walled infrabony wound was filled with macroporous beta-tricalcium phosphate (β-TCP). For the control group, only the PGA sheets were placed. After 6 weeks, histometric analysis demonstrated complete periodontal regeneration with well-oriented collagen fibers connecting the new cementum with new bone. Furthermore, ligament-like tissues were formed near the new bone and cementum, which were further confirmed to consist of collagen fibers [94]. The in vitro-expanded autologous cells were effective for tissue regeneration and had already been employed in several clinical trials, including periodontitis therapies [95]. 

In order to elucidate the ability of nanomaterials on the cementogenic differentiation of stem cells and the mechanisms, the effects of mnHA (hydroxyapatite bioceramics with the micro-nano-hybrid surface) on the attachment, proliferation, cementogenic and osteogenic differentiations of PDLSC were investigated [96]. The mnHA bioceramics promoted cell proliferation, ALP activity, and expression of osteogenic and cementogenic-related markers including CAP and CEMP1, Runx2, ALP, OCN. Moreover, the stimulatory effect on ALP activity and osteogenic and cementogenic gene expressions could be repressed by canonical Wnt signaling inhibitor dickkopf1 (Dkk1). These results demonstrate that mnHA could be promising grafting materials for bone and cementum repair. However, future in vivo investigation is needed to evaluate and verify the efficacy in periodontal regeneration.

Therefore, the cementum-specific proteins had the ability to adjust the periodontal homeostasis and regeneration of cementum structures. Current researches showed that the following novel approaches all promoted the repair and regeneration of cementum: (1) The differentiation of cementoblast progenitors themselves, (2) co-culture with the secreted cytokines by other specific cells, (3) the application of cell sheet, (4) materials with tailored micro-nano-hybrid surfaces. The methods represent the new and better therapeutic approaches for more effectively treating periodontitis and inducing periodontal regeneration. 

## 5. Endogenous Cell-Homing and Host-Mobilized Stem Cells

**Figure 5:**
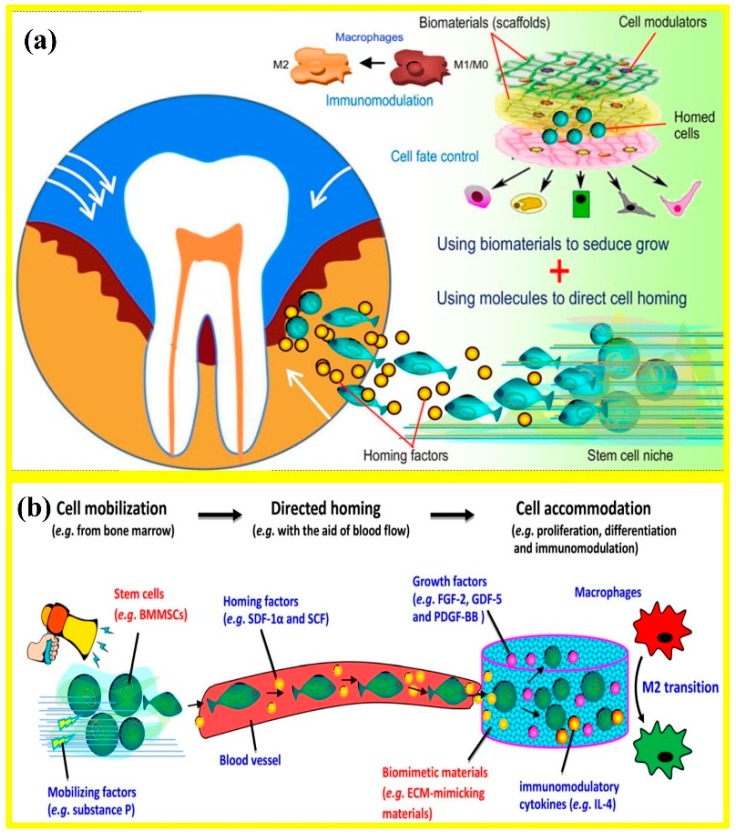
Harnessing endogenous stem cells. (**a**) Periodontal regeneration could be achieved via harnessing endogenous stem cells from the host tissues, where biomaterials and molecules coax the recruitment of endogenous stem cells to induce growth. (**b**) Schematic showing the mobilization of stem cells from their niche using cell-mobilizing factors, directing cell migration using cell homing factors and blood flow, then regulating stem cell fate via materials, growth factors and immunomodulatory cytokines. BMMSCs: bone marrow- mesenchymal stem cells; ECM: extracellular matrix; FGF-2: fibroblast growth factor-2; GDF-5: growth/differentiation factor-5; IL-4: interleukin-4; PDGF-BB: platelet-derived growth factor-BB; SCF: stem cell factor; SDF-1α: stromal-derived factor-1α (adapted from Reference [13], with permission).

Endogenous cell-homing and mobilization of resident stem cells are the basis for endogenous regenerative medicine (ERM). Periodontal regeneration could be achieved via the in vivo manipulation of the cell-material interplay at the defect site, where biomaterials and biomolecules could coax the recruitment of endogenous stem cells to regenerate new tissues (Figure 5a) [13]. This could be achieved via the mobilization of stem cells from their niche using cell-mobilizing bio-factors including substance P and directing the cell migration via blood flow and cell-homing agents such as stromal-derived factor 1α (SDF-1α) and stem cell factor (SCF). In addition, the stem cell fate could be regulated once they reached the wound, usually through the design of biomaterials including the synthesis of ECM-simulating materials and by providing a number of growth factors. These growth factors included fibroblast growth factor-2 (FGF-2), growth/differentiation factor-5G (GDF-5), and platelet-derived growth factor-BB (PDGF-BB) and interleukin-4 (IL-4) (Figure 5b) [13]. The harnessing of endogenous cells for regeneration applications included the following methods [97]: Regeneration via the patients’ own cells [98], coaxing endogenous cell-homing via chemokines [99], and facilitating endogenous cell-homing through materials design [100].

ERM showed great potential in periodontal research due to the high incidence rate of periodontitis, and the mounting evidence showing that endogenous stem cells could be directed to the periodontium to play important regenerative and immune-modulating functions [97]. The innovative and anatomically-shaped human molar scaffolds and rat incisor scaffolds were produced via 3D bioprinting by using a composite of poly-ε-caprolactone (PCL) and HA containing interconnected channels with about 200 µm diameters [101]. An incisor scaffold was placed in vivo orthotopically following mandibular incisor extraction using a rat model, whereas a human molar scaffold was placed ectopically in the dorsum. SDF1 and bone morphogenetic protein-7 (BMP7) were delivered in the scaffolds. Indeed, a PDL-like connective tissue (42%) and new bone were generated at the interface of the rat incisor scaffold with the native alveolar bone, which contained spindle-shaped cells, capillaries and well-organized fibrous collagen in the interstitial spaces. The delivery of SDF1 and BMP7 not only helped recruit many more endogenous cells but also enhanced the angiogenesis when compared to the control scaffolds without growth factors. Therefore, the regeneration of tooth-mimicking structures and periodontium integration via cell-homing provided an alternative to cell delivery [58]. 

Further studies are needed to investigate how to take advantage of the body’s innate regenerative capability and to regulate cell-homing, thereby to enhance the clinical use of the host stem cells for periodontal treatments [102,103].

## 6. 3D-Bioprinting and the Generation of the Oriented PDL Fibers

**Figure 6:**
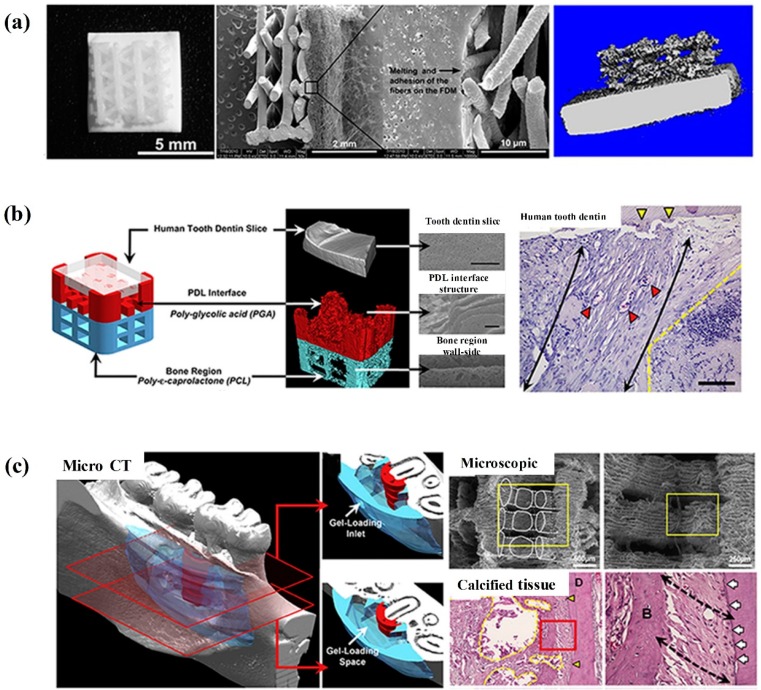
3D-bioengineered periodontal complex. (**a**) Biphasic scaffold with cell sheets. Fabrication of biphasic scaffold and cross-section of the biphasic scaffold by scanning electron microscope (SEM) indicating the fusion of electrospun fibers with fused deposition modeling (FDM) component. Microcomputedtomography (micro-CT) of biphasic osteoblast-seeded scaffold 8 weeks after implantation in a subcutaneous athymic rat model; (**b**) 3D hybrid scaffold. Micro-CT and 3D-reconstructed hybrid scaffold (scale bar = 50 μm). SEM analysis of each layer. Perpendicular orientation of PDL-like tissues to dentin and along the column-like structures (scale bar = 50 μm). (**c**) Bone-ligament complexes with fiber-guided scaffolds. Micro-CT mandibular pictures and customized defect-fit scaffolds. Microscopic architectures of the fiber-guiding scaffold with micro-grooves and macropores (scale bar = 500 and 250 μm) and organized fibrous tissues with mineral tissue layers (adapted from References [107,108,109], with permission).

The defects in periodontal diseases vary in sizes from small intra-bony defects to large horizontal and vertical bone defects. Therefore, efforts were made to achieve predictable and reliable regeneration using bio-fabrication of scaffolds [104,105]. A novel approach produced multi-compartments in PCL via 3D-printing using a fused filament fabrication technique [21,106]. As shown in Figure 6a, a biphasic scaffold was designed to regenerate the PDL compartment, which was comprised of a fused deposition modeling (FDM) scaffold (mimicking the bone compartment) and an electrospun membrane (mimicking the PDL compartment). This was then combined with multiple PDLSC sheets to regenerate the complex periodontal structure. In vitro test indicated that the osteoblasts synthesized a mineral matrix in the bone compartment after culturing for 21 days. The PDL cell-sheet harvesting did not compromise the cell viability. Then, the cell-seeded biphasic scaffolds were put on a dentin block and placed subcutaneously in vivo for 8 weeks using an athymic rat model. Mineral cementum-mimicking tissue was indeed formed on dentin for the scaffolds having multiple PDL cell sheets. Therefore, the combination of multiple PDL cell sheets and a biphasic scaffold simultaneously delivered the cells needed for in vivo generation of alveolar bone, PDL, and cementum [107].

Furthermore, 3D-bioprinting was an effective approach to reconstruct the spatiotemporal orientation PDL fibers [108]. A PCL-PGA fiber-guiding scaffold, consisting of both bone-specific and PDL-specific polymer compartments, were used to engineer tooth-ligament interfaces. This method successfully regenerated the bone and PDL complexes especially the parallel and obliquely oriented fibers [109]. In another study, a 3D printing method was used to produce wax molds, and polymers were cast with PGA in the PDL interface and PCL in the bone architecture [110]. To simulate the periodontal microenvironment, human dentin was used with the dentinal tubules being exposed. Then, an ectopic periodontal tissue regeneration model was constructed in immunodeficient mice using the human dentin slice, PGA-cast PDL scaffold, and PCL-cast bone architecture. This subcutaneous investigation yielded a number of key results: (1) fibrous tissue was generated on the PGA structures with perpendicular or oblique orientations to dentin; (2) mineral tissues were generated in a bone construct and dentin surface, mimicking bone and cementum-like tissue regeneration; (3) ligament-bone tissues were formed with specific, highly controllable compartmentalization and organization. The most important results were the geometric or architectural promotion cues to accurately and predictably control connective tissue orientations in the 250 to 300 µm interfaces. Besides the fibrous tissue orientation, the limited infiltration of the new bone into the PDL architecture enhanced the spatiotemporal tissue organization (Figure 6b) [108].

The fiber-guiding scaffold had several levels of compartmentalization, consisting of a PDL interface with perpendicularly-oriented architecture to the tooth-root surface topography, and a bone area with open structures, possessing macroporosity for tissue ingrowth [21]. The implanted fiber-guiding scaffolds guided the tooth-supportive structure formation, and bone regeneration proceeded into the bone region of the fiber-guiding scaffold and PDL orientation. The cementogenesis occurred on the tooth roots. Besides multiple tissue generation and fibrous connective tissue angulation, the ligamentous tissues were integrated with the mineral tissues, and these complicated structures restored the tooth-supportive function (Figure 6c) [109,110]. Therefore, 3D imaging and modeling technologies could be used to have a great impact to create the “bioactive scaffolding systems” for periodontal regeneration.

## 7. Gene Therapy-Based Approaches for Periodontal Regeneration

**Figure 7:**
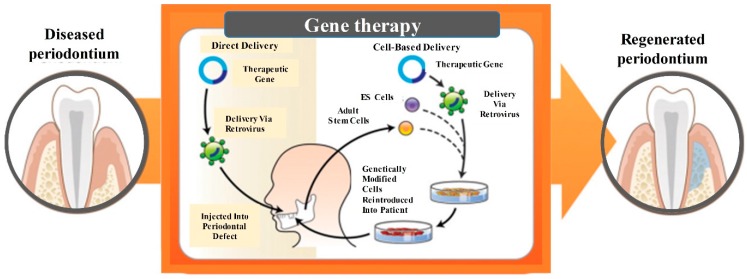
Gene-based technologies. Direct and cell-based delivery of a therapeutic gene increased the regenerative capability and promoted the availability of key bio-factors. The gene of interest was either injected directly into the periodontal defect via a retrovirus, or alternatively, was incorporated into embryonic stem cells (ES) or adult stem cells that were then expanded and delivered into the wound (adapted from Reference [113], with permission).

Gene therapy refers to the treatment of disease by transferring genetic materials to introduce, suppress, or manipulate specific genes that direct an individual’s own cells to produce a therapeutic agent [111]. The foreign gene is injected into the patient by viral or non-viral vectors during in vivo gene transfer. Alternatively, the foreign gene is transduced into the cells of a tissue biopsy outside the body, and then the resulting genetically-modified cells are transplanted back into the patient [112]. For periodontal repair, the gene could be either injected directly into the periodontal defect via a retrovirus or incorporated into embryonic stem cells (ES) or adult stem cells, which were expanded and delivered into the defect (Figure 7) [113]. 

An advantage of gene therapy was that it could achieve greater bioavailability of growth factors within the periodontal wounds [114]. The delivery methods and DNA vectors in periodontal tissue engineering had the goal to maximize the duration of growth factors, optimize the delivery technique to the periodontal wounds, and minimize the patient risk [115]. Indeed, the regeneration of the alveolar bone-PDL-cementum complex in rats was achieved by using localized, controlled PDGF delivery via gene therapy. Periodontal lesions (3 × 2 mm in size) were treated with a 2.6% collagen matrix alone or with a matrix containing adenoviruses encoding luciferase (control), a dominant negative mutant of PDGF-A (PDGF-1308), or PDGF-B. Block biopsies were obtained at 3, 7, and 14 days after gene delivery. The defects treated with Ad-PDGF-B had much more proliferation and more bone and cementum formation than control. Quantitative image analysis demonstrated a 4-fold increase in bone bridge and a 6-fold increase in tooth-lining cementum repair in the Ad-PDGF-B-treated sites, compared with lesions having Ad-luciferase or collagen matrix alone. Therefore, gene therapy using PDGF-B offered exciting results for periodontal tissue engineering applications [116].

Several other strategies of gene therapy in healing and preventing periodontal diseases were reported [117]. One study examined the effects of oral coimmunization of germfree rats with two *Streptococcus gordonii* (*S. gordonii)* recombinants expressing N- and C-terminal epitopes of *Porphyromonas gingivalis (P.g)* FimA to elicit FimA-specific immune responses. The effectiveness of immunization in protecting against alveolar bone loss following *P.g* infection was also evaluated [118]. The results showed that the oral delivery of *P.g* FimA epitopes via *S. gordonii* vectors resulted in the induction of FimA-specific serum (immunoglobulin G (IgG) and IgA) and salivary (IgA) antibody responses. Furthermore, the immune responses were protective against subsequent *P.g*-induced alveolar bone loss, which supported the potential usefulness that gene therapeutics-periodontal vaccination could be exploited to provide protection against *P.g* [118]. Therefore, novel genetic techniques are exciting approaches and expected to receive growing recognition for enhancing the periodontal regeneration in dental practices. 

## 8. Regenerating Bone-PDL-Cementum Complex via Layered Materials and Cells

The periodontium exhibited a typical “layer-by-layer or (LBL) structure” that comprised cementum, alveolar bone and PDL [68]. The PDL consists of highly organized fibers, which are perpendicularly inserted into the cementum-coated tooth root and adjoining the alveolar bone, where their ends (Sharpey’s fibers) insert into the mineralized tissues to stabilize the tooth root, transmit occlusal forces, and provide the sensory function. The complete regeneration of this complex apparatus is very difficult to achieve through the local administration and simple combination of in vitro-cultured stem cells and scaffolds [119]. Inspired by its anatomical structures, the regeneration of the periodontal complex could benefit from specific layered cell-material designs, which consist of different layers containing specific materials, cells and growth factors to recreate the native bone-PDL-cementum complex [94]. 

Does the regeneration of bone, PDL and cementum happen simultaneously or in a sequential manner? A tri-layered scaffold was used for the regeneration of cementum, PDL and bone [11]. The gene expression related to cementum, PDL and bone-related proteins were detected on 7, 14 and 21 days, respectively. These proteins began to express in different degrees from the 7th day, which increased with time. However, the expression of osteogenic gene RUNX2 was significantly higher on the 7th day than other genes. Therefore, it was speculated that the osteogenic process might precede the differentiation of cementum and fibers. At 1 month, the expression of PLAP1 (fibrogenic gene), CEMP1 (cementogenic gene), OCN (osteogenic gene) were all observed at the cementum–PDL–bone interface in the tri-layered group. New cementum, fibrous PDL, and focal areas of new woven bone with a disorganized matrix were observed at the defect site. At 3 months, dense CEMP1, PLAP1 and OCN expressions were all more pronounced in the experiment group. New cementum had cementoblasts aligned along the whole root surface. New fibrous PDL formed by the action of fibroblasts, which was intact and attached to the new cementum and alveolar bone on both sides. New alveolar bone formed with well-defined bony trabeculae. Therefore, the regeneration of bone, PDL and cementum likely occurred simultaneously, although it was also possible that the osteogenic process may slightly precede the differentiation of cementum and fibers [11].

**Figure 8:**
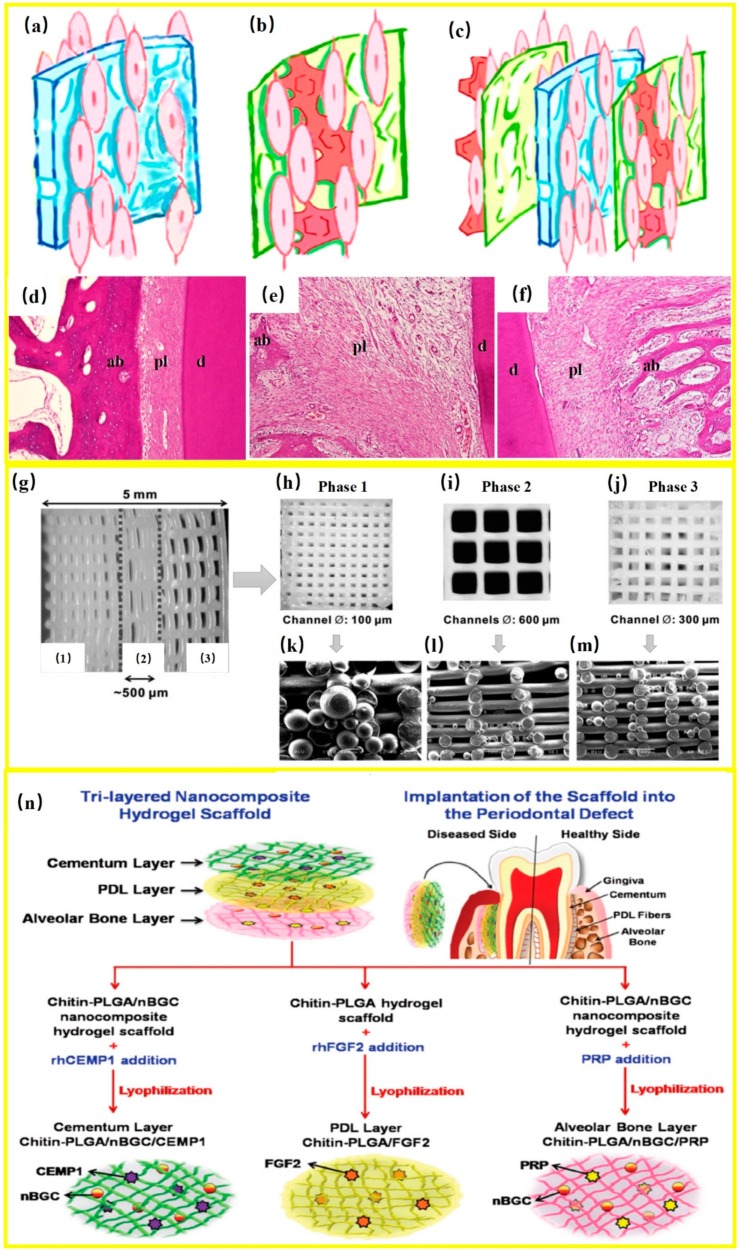
Layered materials and cells to simulate the multiple tissue layers. (**a**) An engineered membrane (Bio-Gide collagen membrane seeded with cells on both sides). (**b**) Two mineralized membranes (a cellular small intestinal submucosa in which cells were seeded on one side and cultured in mineralization-induction medium for 8 days). (**c**) An layer-by-layer (LBL) complex was produced by placing a cell-seeded periodontal membrane between the two cell-seeded/mineralized membranes. (**d**) The LBL group showed that regular alveolar bones and cementum had reconstructed completely, with mature periodontal ligaments and a normal periodontal gap. (hematoxylin and eosin, 100×). (**e**) The trauma group showed disordered fibroblasts and external resorption of teeth, with only a few irregular new bones. (hematoxylin and eosin, 100×). (**f**) The Bio-Gide collagen membrane group showed some new thin and irregular bones; periodontal fibers were disordered, and the periodontal gap was very wide (hematoxylin and eosin, 100×). (**g**) 3D-printed seamless scaffold with region-specific microstructure and spatial delivery of proteins. The scaffold had three phases: (**h**) Phase 1: 100 μm microchannels with 2.5 mm in width, (**i**) Phase 2: 600 μm microchannels with 500 μm in width, (**j**) Phase 3: 300 μm microchannels with 2.25 mm in width. (**k**–**m**) Poly lactic glycolic acid (PLGA) microspheres encapsulating amelogenin, connective tissue growth factor (CTGF), and bone morphogenetic protein 2 (BMP2) were spatially tethered to Phases 1, 2 and 3, respectively. (**n**) Schematic of a tri-layered nanostructured composite hydrogel scaffold (each layer had different growth factors) for simultaneous regeneration of multiple periodontal tissues. ab—alveolar bones; pl—periodontal ligaments; d—dentin; CEMP1: cementum protein-1; FGF-2: fibroblast growth factor-2; PRP: platelet-rich plasma; nBGC: nanobioactive glass ceramic; rhCEMP1: recombinant human cementum protein-1; rhFGF: recombinant human fibroblast growth factor (adapted from References [11,13,120,121], with permission).

Recently, an LBL-like complex was constructed for periodontal regeneration [120]. Gingival fibroblasts (0.5 mL of a 2 × 10^6^ cells/mL solution) were seeded on both sides of the Bio-Gide collagen membrane (5 × 10 mm) and were cultured for 3 days to construct a tissue-engineered periodontal membrane (Figure 8a). At the same time, the cells were also seeded on one side of the small intestinal submucosa (SIS) and cultured in a common medium for 3 days, then in mineralization-induction medium for another 8 days to construct a tissue-engineered mineralized membrane (Figure 8b). The LBL tissue-engineered complex was constructed by placing a tissue-engineered periodontal membrane between two mineralized membranes (Figure 8c). After implanting the complex into the periodontal defect areas (2-mm periodontal tissue defect in the mesial and distal surface of each experimental mesial root) of six dogs, histological observations on the 10th day showed that new alveolar bone and cementum had formed in the LBL group. In addition, new PDL was rich with cells and new blood vessels, and perforating fibers in the alveolar bone and cementum were clearly visible. On the 20th day, the LBL group showed completely reconstructed regular alveolar bone and new cementum, with a mature PDL. The alveolar bone included a cribriform plate and cancellous bone (the trabecular bone and bone marrow). The periodontal fibers were arranged from the cementum to the alveolar bone, forming a 45-degree oblique upward angle with clear Sharpey’s fiber in the alveolar bone and cementum, thus restoring the periodontal gap (Figure 8d). In contrast, the trauma group showed disordered fibroblasts, and only a few irregular new bone regions. Several teeth showed external resorption (Figure 8e). The Bio-Gide collagen membrane group showed newly-formed thin and irregular bone; the periodontal fiber was disordered, and the periodontal gap was very wide (Figure 8f). The new periodontal attachment achieved complete periodontal reconstruction in a relatively short time period in the LBL group. This indicated that the periodontal attachment and complete periodontal reconstruction could be achieved in a short time period in the LBL group. Therefore, the repair effect of the LBL tissue-engineered complex on periodontal defects was markedly superior to that of a single tissue-engineered periodontal membrane.

With computer-aided design and 3D printing techniques, efforts were made to develop new multiphase and region-specific micro-scaffolds with spatiotemporal delivery of bioactive cues for integrated periodontium regeneration [121]. Polycarprolaction chydroxylapatite (90:10 wt%) scaffolds were manufactured using 3D printing seamlessly in three layer-by-layer phases (Figure 8g): 100 μm microchannels in Phase 1 designed for the cementum/dentin interface (Figure 8h), 600 μm microchannels in Phase 2 designed for the PDL (Figure 8i), and 300 μm microchannels in Phase 3 designed for alveolar bone (Figure 8j). Recombinant human amelogenin (Figure 8k), connective tissue growth factor (CTGF) (Figure 8l), and BMP2 (Figure 8m) were spatially delivered and time-released in Phases 1, 2, and 3, respectively. Upon 4-week in vitro incubation separately with DPSCs, PDLSCs and alveolar bone stem/progenitor cells (ABSCs), specific tissue phenotypes were obtained with collagen I-rich fibers by PDLSCs and mineral tissues by DPSCs, PDLSCs, and ABSCs. During in vivo implantation, the DPSC-seeded multiphase scaffolds generated new PDL-mimicking collagen fibers that were attached to the new bone and dentin/cementum tissues.

In addition, tissue structure-simulating materials were combined with growth factors, antibodies, and drugs to enhance the creation of a feasible local microenvironment and promote cell-homing and tissue formation [103,122]. For the concurrent formation of the three types of periodontal tissues, each layer was specifically designed to contain chitin-PLGA and/or nanobioactive glass ceramic (nBGC) components [11]. The layer with chitin-PLGA/nBGC was loaded with recombinant CEMP1 to form new cementum. Similar components were combined with platelet-rich plasma (PRP) to form new bone. A chitin-PLGA hydrogel was loaded with recombinant human FGF-2 for PDL regeneration. Thus, a tri-layered nanostructured composite scaffold was fabricated by assembling chitin-PLGA/nBGC/CEMP1, chitin-PLGA/FGF2, and chitin-PLGA/nBGC/PRP layers in the order of periodontal tissues (Figure 8n). In vivo, the tri-layered nanocomposite hydrogel scaffold with/without growth factors was placed in the rabbit maxillary periodontal defects. The tri-layered nano-scaffold with growth factors achieved full defect closure and healing, with new cancellous-like tissue as shown in microcomputed tomography analyses. Histological and immunohistochemical analyses verified the generation of new cementum, fibrous PDL, and alveolar bone containing well-defined bony trabeculae. Therefore, the cell-free scaffold was applied to periodontal defects and achieved the full regeneration of the tissues in the periodontium by enhancing host cell-homing [13].

Compared to the LBL apparatus, the computer-aided design and 3D printing of the new multiphase, region-specific, micro-scaffolds had a higher precision. Furthermore, the tissue structure-mimicking biomaterials with the ability to promote cell-homing avoided the need for transplantation of exogenous cells. These advances indicate that the structure and function of all the destroyed periodontal tissues (bone-PDL-cementum) could be completely and synchronously restored, via cementogenesis, osteogenesis and the formation of periodontal ligaments.

## 9. Conclusions

This article represents the first systematic review on new developments in regenerating all three types of periodontal tissues and the simultaneous regeneration of the entire bone-PDL-cementum complex, via stem cells, 3D-printing, gene therapy, and layered bio-structures. Four novel approaches were investigated for periodontal regeneration. (1) Cell-homing aimed to avoid the ethical problems of using embryonic stem cells, and to prevent the potential immune rejection in transplanting exogenous cells. (2) 3D-printing yielded novel scaffolds more quickly and precisely, with tailored biomimetic structures and bioactive compositions. (3) Gene therapy could precisely target the regulation of the microenvironment of the defect to enhance periodontal regeneration. (4) Combinational methods were developed to regenerate the complex structure of the bone-PDL-cementum apparatus by designing layered materials and cells to biomimetically regenerate periodontal structures completely and synchronously. Currently, several types of bone grafts such as FDBAs, DFDBAs, Bio-Oss, collagen membrane, and several 3D-bioprinted materials, have been approved and commercialized for clinical use in patients. The use of DPSCs and several other types of stem cells are currently in the clinical trial stage. The cell-homing, gene therapy and layered materials for periodontal regeneration are still in the laboratory research stage. These new researches on stem cells and novel scaffolds have the potential to greatly enhance the periodontal bone-ligament-cementum regeneration efficacy, as well as to impact other areas of tissue engineering and regenerative medicine.
